# Ways of coping with premenstrual change: development and validation of a premenstrual coping measure

**DOI:** 10.1186/1472-6874-14-1

**Published:** 2014-01-03

**Authors:** Jennifer R Read, Janette Perz, Jane M Ussher

**Affiliations:** 1 Centre for Health Research, University of Western Sydney, Locked Bag 1797, Penrith, NSW 2751, Australia

**Keywords:** PMS, Premenstrual change, Premenstrual coping, Scale development, Coping

## Abstract

**Background:**

Negative premenstrual change can result in distress for a significant proportion of women. Previous research has suggested that women employ a range of coping strategies and behaviours in order to manage and reduce premenstrual distress. However, as yet there has been no specific scale available to measure premenstrual coping. This research aimed to develop and validate a measure of premenstrual coping which can be used in future investigations of negative premenstrual experience.

**Methods:**

A sample of 250 women living in Australia, reporting mild to severe premenstrual distress, completed an online survey containing 64 items related to premenstrual coping. The items were generated by reviewing past literature related to premenstrual experience, in particular recent qualitative research on premenstrual coping. A principal components factor analysis with varimax rotation was conducted to determine item clusters that would form a measure. Reliability and validity were tested using calculations of Cronbach alphas, correlational analysis with psychological coping scales and a content analysis of participant reports of coping strategies.

**Results:**

The factor analysis, which involved two principal component analyses, resulted in five factors containing 32 premenstrual coping behaviours. Interpretation of the factor solution drew on empirical and theoretical accounts of premenstrual coping and the emergent factors were labelled Avoiding Harm, Awareness and Acceptance of Premenstrual Change, Adjusting Energy, Self-Care, and Communicating. These factors form the subscales of the Premenstrual Coping Measure (PMCM). The subscales demonstrated acceptable to very good reliability and tests of construct, concurrent and content validity were supportive of sound validity.

**Conclusions:**

The PMCM provides a valid and reliable scale for quantifying ways of coping specific to negative premenstrual change. Conceptual similarity was found between some coping behaviours and behaviours positioned as symptoms of premenstrual change. Explanations for this overlap may be found in cultural discourses associated with idealised femininity and PMS (premenstrual syndrome). Further psychometric investigation of the PMCM will enhance knowledge of the role of coping with negative premenstrual experience.

## Background

Premenstrual change is experienced by 90-95% of women of reproductive age [1,2]. Over 300 different premenstrual changes have been reported, with the most common being irritability, headaches, depression and tension [3,4]. Other frequently experienced changes include fatigue, mood swings, anxiety, breast tenderness and difficulty concentrating [5]. Approximately 40% of Western women are estimated to report moderate distress associated with these changes, described as premenstrual syndrome (PMS) [6,7], and between 3-8% are estimated to experience severe premenstrual distress [8,9], described as premenstrual dysphoric disorder (PMDD). The magnitude of the impact of negative premenstrual change demonstrates that this is an important topic for investigation in the field of women’s health.

Management of moderate-severe premenstrual distress has traditionally focused on medical treatments, developing from irradiation of the ovaries [10], to serotonin reuptake inhibitors (SSRIs), as the primary pharmacological option [8,11]. This type of treatment assumes a purely biomedical view of premenstrual experience, positioning PMS and PMDD as fixed pathologies within the woman, caused by hormonal or neurotransmitter imbalance [12]. However, there is growing evidence that psycho-social factors are associated with the development and course of premenstrual symptoms [6,13], and that psychological interventions are effective in reducing premenstrual distress [14-16]. For example, cognitive restructuring skills taught as part of Cognitive Behaviour Therapy (CBT), have been found to be effective in reducing cognitive, affective and somatic premenstrual symptoms [17-21]. Training in the use of active behavioural coping strategies, which include exercising, going out with friends and keeping busy, has also been found to result in better outcomes [20]. This suggests that attention should be paid to the factors that facilitate women’s ability to cope with negative premenstrual change.

The contextual cognitive model of coping, views coping as a manner of thinking, feeling and behaving that arises in a situation that an individual appraises as significant and difficult [22]. The premenstrual phase of the cycle is a time when many women are vulnerable to negative appraisals, because of dominant cultural discourses that position the premenstrual women as “mad, bad or dangerous” [6]. In this vein, research has demonstrated that women make internal embodied attributions for negative emotions experienced premenstrually, whereas they make external attributions for the same emotions at other times in the cycle [23,24]. There is also evidence that women experience increased sensitivity to emotions, or to external stress, during the premenstrual phase of the cycle [25-29]. Some women report that sensory perception is more acute premenstrually [13], which can result in environmental stress being experienced as more challenging [30], and the responsibilities which are a normal part of most women’s lives being experienced as more burdensome [31,32]. Emotions such as anger, sadness or irritability can also be experienced as more potent than usual [31,33]. This has led to the suggestion that the premenstrual phase of the cycle is a stressor in and of itself [26], with the severity of premenstrual distress influenced by women’s style of coping [34].

Previous research on premenstrual coping has examined the relationship between ways of coping and premenstrual symptom severity [35,36], coping at different phases of the menstrual cycle [27,28], and the association of coping with other psychological characteristics, including depression and anxiety [36]. There are indications from this research that women use less cognitive restructuring when they are premenstrual [29,36], and that women who cope by *distancing* themselves from stress experience lower premenstrual symptom severity [37]. Distancing involves not becoming overly focused on the stressor, being detached, and accepting the situation [38]. *Structuring*, which refers to coping by utilising resources such as time and energy, by planning and setting appropriate limits, has also been associated with reports of lower severe premenstrual symptoms [39]. It has been suggested that women’s coping strategies vary across the menstrual cycle, with emotion focused coping increasing, and task avoidance, as well as social diversion-oriented coping, decreasing premenstrually [39]. Conversely, it has been reported that women who report higher rates of depression premenstrually are more likely to engage in avoidant coping, independent of cycle phase [36]. Emotional and material support from family and friends has also been associated with reports of lower premenstrual symptoms, and more effective coping [35,40,41].

However, the focus of this previous research has been on women’s strategies of coping with life stressors rather than coping with premenstrual change itself; coping in these studies has thus been measured using generic coping scales, rather than scales specific to premenstrual coping. This has led to the suggestion [42] that specific coping scales for premenstrual change need to be developed. Folkman [43] suggests that qualitative approaches can be useful in uncovering specific ways of coping in a particular context, which can then be used to develop quantitative measures. Recent qualitative research [34] involving interviews with women reporting moderate to severe PMS, revealed that women use a range of coping strategies to reduce or avoid distress. These included anticipation and planning, avoiding stress, self-care, solitude, not expressing anger or irritation, seeking social support and taking supplements or drugs. This study also found that women who were more aware and accepting of premenstrual changes were more likely to implement these coping strategies. Another recent study of premenstrual experience [44], which used open ended questions in a written questionnaire, found that the ways of coping varied according to the type of change experienced. Consuming sweets, sleeping, resting, having showers and taking pain killers were the most commonly identified coping strategies in this research. In combination, these strategies could form the basis for the development of items in a premenstrual coping scale.

Development of a context specific premenstrual coping scale would allow researchers to deepen their understanding of the nature and effectiveness of strategies of coping associated with negative premenstrual change and distress. Being able to evaluate premenstrual coping in a quantifiable manner may also help facilitate the evaluation of interventions to reduce premenstrual distress. Development of a premenstrual coping measure, therefore, has both research and clinical relevance. This research aimed to develop a psychometrically sound measure of premenstrual coping so that the role of coping in premenstrual experience can be explored more fully.

## Methods

### Participants and procedure

A sample of 250 women participants, aged between 18 and 49 years who experienced regular menstrual periods, with a menstrual cycle length from 21 to 35 days, and had not been pregnant or lactating in the past 12 months, was recruited using two methods. The first method was an advertisement on the social media website Facebook (http://www.facebook.com) asking for women who experienced PMS, to join a study on coping with premenstrual change. The advertising campaign targeted women in the relevant age group living in Australia. This recruitment approach resulted in responses from 204 women. The additional 95 women were undergraduate psychology students participating in research for course credit. The women were not required to meet formal diagnostic criteria for a premenstrual disorder (PMD) to be included in the study, as a sample with a wide range of premenstrual experiences was deemed desirable for the development of the coping scale.

Only women residing in Australia were recruited for the study, as a participant group living in the same commercial context was desired. Although the use of medications was listed in the item pool for the coping scale, Australia has no direct marketing of pharmaceuticals, including medications targeted at premenstrual distress, which is a factor to consider when examining ways of coping with premenstrual change across different contexts. Of the 299 surveys initiated, 260 were sufficiently completed to be included in the final analysis. Five of these were excluded because the women were outside the age group or living overseas. An additional five were excluded because the women reported having no premenstrual changes.

Two measures were used to assess the participants’ experience of premenstrual change. The Premenstrual Symptoms Screening Tool (PSST) [45], which is a retrospective measure matching symptoms and impairment criteria for PMDD from the Diagnostic and Statistical Manual of Mental Disorders [46] and a one-item scale asking: “To what extent do you find your PMS distressing?” which is a measure that has been developed and used in previous research in the field of PMS [6,20]. The rationale for using the PSST in this study was that it served to identify whether the women were experiencing any negative premenstrual changes. This measure was not used as a diagnostic tool for PMD but served to demonstrate that all women included in the study experienced at least one negative premenstrual change. This measure also helped to describe the sample in terms of the prevalence of PMD.

The recruitment procedure redirected potential participants to the study’s participant information screen, via a paid advertisement on the social media website, or via university websites. Participants indicated consent and willingness to participate by clicking to begin the survey. Participants completed an anonymous 30 minute online survey administered through the commercial survey website, Survey Monkey (http://www.surveymonkey.com). The survey consisted of some initial questions regarding demographic information followed by self-report measures for premenstrual change and premenstrual coping. The research protocol was approved by the University of Western Sydney Human Research Ethics Committee.

### Premenstrual coping item pool

The initial item pool for the PMCM was developed with awareness of the need to ensure a high level of content validity. Firstly, a review of the coping literature in general highlighted the ways in which coping is commonly measured. Although there is no consensus on a definitive list of ways of coping, analysis of numerous coping scales by Skinner et al. [47] has allowed the identification of common coping strategies. These include problem solving, support seeking, escape/avoidance, distraction, positive cognitive restructuring, social withdrawal, emotional regulation, rumination, helplessness, information seeking, negotiation and opposition. Secondly, a review of the literature on coping with premenstrual change was conducted to identify coping strategies specific to the context of premenstrual experiences. Items were then written based closely on narrative accounts of coping found in the literature. These items were then grouped into a number of conceptual categories of coping which were derived from looking at the themes present in the item pool. The final item pool of 64 items was created from this list, with the nine categories being *Looking After the Body, Planning, Self-Care, Social Support and Communication, Avoiding Harm to Self, Acceptance, Awareness, Emotional Regulation* and *Desire to Be Alone.*

The category of items called Looking After the Body included items regarding diet, exercise, sex, supplements and both over the counter and prescription drugs. Planning items were concerned with women tracking their menstrual cycle. Self-care items included a range of activities where the woman focuses on her own needs and wellbeing. Social Support and Communication items were about communicating feelings and needs with others. Items in the Avoiding Harm to Self category covered behaviours that women might use to avoid thoughts and interactions which held the potential for increased stress. Acceptance items identified an attitude of acceptance of premenstrual change as a normal part of a woman’s experience and an absence of negative appraisals of the changes. Awareness items included items regarding awareness of physical and emotional premenstrual changes as well as awareness of the need to employ coping strategies. A range of Emotional Regulation items were included which gave options for both controlling and expressing feelings as ways of coping. The Desire to Be Alone items reflected research indicating that social withdrawal is an adaptive coping strategy for women during the premenstrual period. This original item pool is displayed in Table 1.

**Table 1:** Original item pool for the premenstrual coping measure

**Category**	**Item**
**Looking after the body**	I make more of an effort to eat healthy food
	I eat less sugary foods
	I eat more sugary foods
	I increase my alcohol consumption
	I decrease my alcohol consumption
	I increase my sexual activity
	I decrease my sexual activity
	I exercise more
	I exercise less
	I take vitamins or minerals
	I use alternative forms of treatment e.g. naturopathy, acupuncture,
	I take prescribed medication
	I take over the counter medicine for pain relief e.g. paracetamol
**Planning**	I refer to a calendar or diary to know when I will be premenstrual
	I use a mobile app or online tool to track when I will be premenstrual
**Self-care**	I allow myself extra time to rest
	I do things to make myself more comfortable
	I take time to focus on my own needs
	I focus less on the needs of others
	I make time to do things that I enjoy
	I spend time doing things that help me relax e.g. have a bath, massage, read a book
	I take time out from my usual responsibilities
	I give myself permission to let go of the usual demands I place on myself
**Social support and communication**	I tell others about how I am feeling
	I ask for help from others
	I feel confident to tell people how I feel
	I feel confident to tell people what I need
**Avoiding harm to self**	I avoid thinking about things that I know annoy me
	I avoid having conversations that are liable to upset me
	I avoid raising topics that have the potential to create conflict
	I raise issues that I usually keep to myself
	I try to avoid dealing with difficult family issues
	I avoid people that have the potential to provoke me
	I avoid situations that have the potential to provoke me
	I avoid situations where I know I will feel vulnerable
	I remove myself from a situation if it starts to provoke me
**Acceptance**	I believe that my premenstrual changes are a normal part of a woman’s experience
	I know that other women go through this
	I see positive aspects to my premenstrual change
	I think it is okay to be feeling differently when I am premenstrual
	I think it is okay to be more emotional or sensitive when I am premenstrual
	I think it is okay that my physical needs may be different when I am premenstrual
	I think that what I feel like doing shouldn’t change when I am premenstrual
	I accept my changeable moods
**Awareness**	I am aware of my bodily changes
	I am aware of my emotional changes
	I am aware that my premenstrual changes are only temporary.
	I am aware of things that ‘trigger’ me when I am premenstrual
	I know what I need to do to support myself
	I know when I am beginning to feel ‘premenstrual’
**Emotional regulation**	I try to keep my emotions under control
	I openly express increased feelings of anger, frustration or irritability
	I vent my feelings through emotional outbursts
	I try not to express how I am feeling
	I don’t express my feelings in the heat of the moment but talk about my feelings later when I feel calmer
	I am able to express my anger or irritation without blaming others
	I use positive self-talk
	I challenge my negative thoughts
**Desire to be alone**	I take time to be on my own
	I enjoy doing things on my own
	I withdraw from others
	I communicate with others about my need to be on my own
	I decrease my social activities
	I increase my social activities

Deriving the item pool from the research literature on premenstrual coping helped to establish content validity in regard to the premenstrual context. Content validity was also assessed in terms of coping strategies in general. The coping processes identified by Skinner et al. [47], and the subscales of the Brief COPE [48], a commonly used generic coping measure, were used as a comparison framework for this assessment. This comparison framework, which is represented in Table 2, revealed that the premenstrual coping item pool encompassed relevant coping processes for this context, supporting further the content validity of the items. One process not contained in the item pool was information seeking. This coping process was not considered relevant for the scale, because although potentially useful, it is unlikely to be used as a regular coping strategy each month. The order of the item pool was randomised prior to administration.

**Table 2:** Coping comparison framework

**Skinner’s analysis of coping**	**Carver’s brief cope**	**Premenstrual coping measure**
Problem solving	Active coping	Looking after the body
	Planning	Planning
	Substance use	
Support seeking	Using emotional support	Social support and communication
	Instrumental support	
Escape avoidance	Denial	Avoiding harm to self
Distraction	Self-distraction	Self-care
Positive cognitive restructuring	Positive reframing	Awareness
	Acceptance	Acceptance
	Religion	
Social withdrawal		Desire to be alone
Emotional regulation		Emotional regulation
Rumination		
Helplessness	Behavioural disengagement self-blame	
Negotiation		Self-care
Opposition	Venting	
Information seeking		

Comparisons of commonly identified coping processes, coping subscales of the Brief COPE and categories of items for the original item pool for the PMCM.

### Assessment of coping

For each coping strategy in the item pool, participants indicated the extent to which each statement applied to their experience of coping with premenstrual change using a 5-point rating scale ranging from *Doesn’t apply to me* to *Almost always applies to me.* In order to enable assessment of concurrent and content validity, the following measures were also administered:

***Dealing with PMS.*** Dealing with PMS was assessed using a one-item measure asking, “To what extent do you feel you can you deal with your PMS?” The response was indicated on a 10-point rating scale, with 1 indicating *not at all* and 10 indicating *very well*. This measure has been used in previous research in the field of PMS [6,20].

***The Brief COPE*** [48]*.* This is a generic coping measure containing 14 subscales, each with two items. Internal consistency reliabilities of the subscales are acceptable with Cronbach’s alphas ranging from .50 to .90 [48]. The instruction given before the list of items determines the context for the Brief COPE, which in this case was “In regard to your premenstrual experience, please indicate the extent you usually do what each item says”. This measure uses a 4-point rating scale ranging from *don’t do this* to *do this a lot*. Examples of items include, “I get emotional support from others” and “I try to come up with a strategy about what to do”.

***Open response items for coping.*** Two open response items were asked to identify the most and least helpful strategies adopted. These items were “What are the three most helpful things you do when you are premenstrual?” and “What are the three least helpful things you do when you are premenstrual?”

### Statistical analysis

#### Psychometric development and testing of the PMCM

An exploratory factor analysis, using principal components analysis with varimax rotation, was conducted to identify the items and structure of the coping item pool [49]. Psychometric properties of the resulting factor solution were then evaluated using a number of tests. Firstly, internal consistency reliability was assessed for each factor using Cronbach alpha scores [50]. Secondly, a test of construct validity using bivariate correlation analyses [51] was conducted between the premenstrual coping factors and subscales of the Brief COPE. Concurrent validity was assessed through bivariate correlation analyses between the coping factors and the one-item scale measuring ability to deal with PMS. A final evaluation of content validity was undertaken through a content analysis comparing the composition of the coping factors with the open-ended responses for premenstrual coping.

## Results

The mean age of the women in the sample was 27.09 years (*SD* = 9.35). Of the total sample, 57% were partnered and 43% were not in a partnered relationship. Ninety-one per cent of the participants identified as heterosexual and 9% as non-heterosexual. 26% indicated that they had dependent children. Seventy-four per cent of the participants were employed and 20% worked 30 hours or more per week. In terms of education level, 33% held or were studying towards a university degree, 25% had achieved a trade or diploma qualification, 42% had completed secondary education and 8% had not completed secondary education. Most of the participants (77%) identified as Anglo-Australian, the remainder identifying as belonging to an Asian (17%) or other (6%) ethnicity. While the psychology student participants lived in the greater metropolitan area of Sydney, the participants recruited through social media lived in all states and territories of Australia with the majority coming from NSW. Twenty-four percent of the women in the sample were taking an oral contraceptive, 12% were taking ongoing pain medication for chronic pain related to illness or injury and 25% reported that they were currently experiencing PMS.

Ratings from the PSST revealed that 58% of the women met criteria for moderate to severe PMS. Evaluations from the one-item measure of PMS distress showed that 73% of the women rated themselves as experiencing moderate to severe distress from PMS.

### Factor analysis

A principal components analysis with varimax rotation was conducted on participant responses to the 64 premenstrual coping items from the item pool. Seventeen components with eigenvalues greater than one were extracted from the initial analysis accounting for 67.76% of the variance. After examination of the scree plot, a second principal components analysis forcing seven factors was performed. The rotation converged after nine iterations. The Kaiser’s measure of sampling adequacy of .87 and a significant Bartlett’s Test, *p* < .001, indicated the structure provided a good fit to the data [52].

Item reduction was then achieved according to the following steps. Firstly, 19 items were deleted for cross loading or not loading above the significant factor loading of .326, given a sample size of 250 [49]. Secondly, 14 items with communalities less than 0.4 were deleted [53], with acceptable communalities found for the remaining items. One exception was made for the item “I eat more sugary foods” which although it had a communality of .366, was retained because of its status as one of the most helpful coping strategies reported in the open response items of the survey. Two of the components contained one and two items respectively, and were discarded on the recommendation that components with such few items should only be retained if sample size exceeds 300 [54].

The final simple structure contained five factors with 32 items explaining a total of 38.98% of the variance. These factors and items were used to form the Premenstrual Coping Measure (PMCM). The PMCM contains five subscales: *Avoiding Harm,* which had eight items*; Awareness and Acceptance of Premenstrual Changes,* which had ten items*; Adjusting Energy,* which had five items*; Self-Care,* which had four items*;* and *Communicating,* which had five items. Examples of items from the subscales include: Avoiding Harm “I avoid situations that have the potential to provoke me”; Awareness and Acceptance of Premenstrual Change “I accept my changeable moods”; Adjusting Energy “I decrease my social activities”; Self-Care “I spend time doing things that help me relax”; and Communicating “I tell others about how I am feeling”. The negative loading of item 31, “I try not to express how I am feeling” indicates that this item requires reverse coding when scoring this subscale. Table 3 presents the varimax rotated component loadings, communalities and variance explained for each of the five subscales.

**Table 3:** **Varimax rotated component loadings, communalities (h**^**2**^**), percentages of variance explained and Cronbach alphas for the PMCM**

**Components and items**	**Loadings**	***h***^***2***^	**Variance explained**	**α**
**Avoiding harm**			11.05	.89
1. I avoid situations that have the potential to provoke me	.816	.764		
2. I avoid people that have the potential to provoke me	.809	.747		
3. I avoid raising topics that have the potential to create conflict	.788	.718		
4. I remove myself from a situation if it starts to provoke me	.765	.699		
5. I avoid situations where I know I will feel vulnerable	.762	.692		
6. I avoid having conversations that are liable to upset me	.733	.661		
7. I try to avoid dealing with difficult family issues	.669	.584		
8. I challenge my negative thoughts	.500	.507		
**Awareness and acceptance of premenstrual change**			8.54	.86
9. I accept my changeable moods	.676	.577		
10. I am aware that my premenstrual changes are only temporary	.670	.496		
11. I think it is okay to be feeling differently when I am premenstrual	.664	.585		
12. I am aware of my bodily changes	.660	.545		
13. I think it is okay to be more emotional or sensitive when I am premenstrual	.658	.632		
14. I am aware of my emotional changes	.655	.634		
15. I think that my premenstrual changes are a normal part of a woman’s experience	.637	.457		
16. I know that other women go through this	.574	.466		
17. I think it is okay that my physical needs may be different	.454	.445		
18. I know what I need to do to support myself	.437	.431		
**Adjusting energy**			7.80	.73
19. I vent my feelings through emotional outbursts	.660	.546		
20. I decrease my social activities	.628	.568		
21. I focus less on the needs of others	.608	.480		
22. I exercise less	.605	.482		
23. I eat more sugary foods	.540	.366		
**Self-care**			6.61	.81
24. I spend time doing things that help me relax e.g. have a bath, massage, read a book	.698	.618		
25. I take time to focus on my own needs	.681	.622		
26. I allow myself extra time to rest	.678	.641		
27. I do things to make myself more comfortable	.647	.576		
**Communicating**			4.98	.68
28. I feel confident to tell people how I feel	.665	.579		
29. I feel confident to tell people what I need	.663	.569		
30. I tell others about how I am feeling	.500	.410		
31. I try not to express how I am feeling	-.470	.394		
32. I ask for help from others	.378	.397		

### Reliability testing of the PMCM

Reliability analysis was conducted for the five PMCM subscales to ascertain their internal consistency reliability as measured by Cronbach’s alpha. The Cronbach alphas, displayed in Table 3, were all in the “respectable” to “very good” range except for the subscale Communicating (α = .68) which is considered “minimally acceptable” [50]. Reliability coefficients are acceptable at this level, if the subscale has less than 10 items and support for its validity [51], both of which apply to the subscale of Communicating. This subscale has four items and achieved sound concurrent validity, supported through correlations with appropriate subscales of the Brief Cope, and content validity when compared to the open response list of most helpful coping strategies, as noted below.

### Validity testing of the PMCM

Bivariate correlational analyses between each of the subscales of the PMCM and the one-item scale measuring ability to deal with PMS were conducted to assess concurrent validity. The subscale of Awareness and Acceptance of Premenstrual Change was significantly positively correlated with being able to deal with PMS, indicating that higher scores on Awareness and Acceptance are associated with a greater perceived ability to deal with PMS. On the other hand, the strategies of Avoiding Harm, Adjusting Energy and Self-Care were significantly negatively correlated with being able to deal with PMS. This demonstrates that higher scores on those coping processes are associated with a lower perceived ability to deal with PMS. The Communicating subscale, although demonstrating a positive correlation to being able to deal with PMS, was not significant. These results, which are presented in Table 4, provide mixed support for the concurrent validity of the PMCM subscales. This table also shows the intercorrelations among the subscales which indicate they were all associated significantly with each other in a positive direction, with *r* ranging from .188 to .518.

**Table 4:** Intercorrelations, means, standard deviations and ranges of the PMCM subscales and the Dealing with PMS measure

**Variable**	**1**	**2**	**3**	**4**	**5**	***M***	***SD***	**Range**
Coping								
1. Deal with PMS	-	-	-	-	-	6.33	2.21	1-10
2. Avoiding Harm	-.272***	-	-	-	-	20.61	7.97	8-40
3. Aware and Accept	.125*	.344***	-	-	-	35.07	7.52	10-50
4. Energy	-.512***	.452***	.216**	-	-	14.35	4.50	5-25
5. Self-Care	-.220***	.518***	.398***	.408***	-	11.36	3.74	4-20
6. Communicate	.073	.209**	.410***	.188**	.378***	13.41	3.63	5-24

*Note.* *p < .05.**p < .01. ***p < .001.

Bivariate correlational analyses between the subscales of the PMCM and the Brief COPE were conducted to assess construct validity. A number of meaningful, statistically significant correlations were evident, giving support to the validity of the new scale. One example of this is the relationship between the Brief COPE subscale of Active Coping and the PMCM subscale of Avoiding Harm, *r* (248) = .30, *p* < .001. Active Coping items involve taking action to make things better, which is conceptually related to some of the Avoiding Harm items which involve taking action to avoid making things worse. Another example, is the relationship between the Brief COPE Emotional Support subscale and the PMCM Communicating subscale *r* (248) = .44, *p* < .001. Emotional Support items refer to obtaining comfort, understanding and support from others which has relevance for the Communicating subscale which is about asking for help from others and telling others how you feel when you are premenstrual. These results are shown in Table 5.

**Table 5:** Intercorrelations of the PMCM and the Brief COPE subscales

**Variable**	**Avoiding harm**	**Awareness and acceptance**	**Adjusting energy**	**Self-care**	**Communicating**
Self-distraction	.288***	.199**	.072	.252***	.233***
Active	.303***	.351***	.150*	.257***	.307***
Denial	.252***	-.128*	.163*	.106	.183**
Substance	.195**	-.049	.202**	-.044	.064
Emotional support	.238***	.251***	.173**	.342***	.441***
Instrumental support	.309***	.141*	.091	.315***	.333***
Behavioural disengagement	.228***	-.143*	.329***	.131*	.086
Venting	.278***	.310***	.227***	.209**	.225***
Positive reframing	.287***	.251***	.004	.291***	.274*
Planning	.243***	.291***	.117*	.225***	.235***
Accepting	.371***	.168**	.215**	.444***	.315***
Religion	.220***	.038	.424***	.106	.171**
Self-Blame	.333***	.133*	.232**	.192**	.131*
Humour	.238***	.183**	.059	.279***	.201**

*Note.* *p < .05.**p < .01. ***p < .001.

Support for content validity of the PMCM was also assessed against the results of a content analysis of the open ended list of most helpful and least helpful strategies. The majority (72%) of the most helpful strategies were reflected in the final 32 items of the PMCM. The majority (65%) of least helpful responses were also related to the items in such a way as to support content validity. For example, many of the things reported to be the least helpful matched items in the Avoiding Harm subscale or were the opposite of items contained in the remaining coping subscales. These findings support both content and construct validity. Examination of the content analysis also revealed that some responses were common to both the most and least helpful ways of premenstrual coping. Examples of these include crying, sleeping and eating. This analysis, and a comparison to the components of the PMCM, is displayed in Tables 6 and 7.

**Table 6:** Comparison of content analysis of most helpful premenstrual coping strategies with the subscales of the PMCM

**PMCM subscale**	**Coded response**	**Number of responses**
Avoiding harm		
	Avoid irritations	13
	Positive thinking	6
Awareness and acceptance		
	Self-aware/awareness of premenstrual change	10
	Acceptance	9
	Keep track of menstrual cycle	6
	Know it is only temporary	8
	Be prepared for period	4
Adjusting energy		
	Time alone/out/for self	36
	Eat sugar/chocolate	27
	Eat	15
	Crying	4
Self-care		
	Rest, relax, slow down	53
	Sleep	42
	Watch TV, movies, read	37
	Hot water bottle/heat pack	28
	Bath/Shower	20
	Do things I enjoy	10
	Music	9
	Massage	8
	Make comfortable	6
	Warm food and drink	5
Communicating		
	Social support/partner support	42
	Express how you feel/communicate with others	17
Non matching codes		
	Pain killers	44
	Exercise	28
	Eat healthy	9
	Distraction	7
	Vitamins	6
	Drink more water	5
	Cook	5
	Spend time with pets	5
	Clean	4
	Shopping	4
	Yoga	4

*Note.* The table indicates the number of women who gave the response as one of the most helpful things they do when they are premenstrual. Items with 3 or less responses have not been included in the table.

**Table 7:** Comparison of content analysis of least helpful premenstrual coping strategies with the subscales of the PMCM

**PMCM subscale**	**Coded response for least helpful things**	**Number of responses**
Avoiding harm		
	Conflict/argue/raise issues with people	33
	Give into moods/emotions	26
	Stress/deadlines	22
	Be with negative/irritating people	18
	Ruminating/over thinking	17
	Take frustrations out on others	16
	Deal with relationship/family issues	9
	Complain	6
Awareness and acceptance		
	Frustrated with/criticise self	10
	Ignoring feelings	8
	Ignoring feelings	8
	Not being aware of being premenstrual	4
Adjusting energy		
	Exercise	21
	Overdoing it e.g. exercising, socialising	12
	Not adjusting	10
	Not enough sleep	10
	Socialising	7
Self-care		
	Work/Study	23
	Eating unhealthy food	17
	Housework	6
Communicating		
	Isolating self	14
Non matching codes		
	Binge eating/overeating	18
	Not control emotions	17
	Drinking alcohol/taking drugs	17
	Eat chocolate/sugar	12
	Increase caffeine	7
	Eating	6
	Sleep	5
	Cry	4
	Forget vitamins	4
	Jokes/comments about PMS	4

*Note.* The table indicates the number of women who gave the response as one of the least helpful things they do when they are premenstrual. Items with 3 or less responses have not been included in the table.

The PMCM provides premenstrual coping subscales scores, but use of a total coping score calculated from these subscales is not recommended. The focus of the measure is on ways of coping, not amount of coping, and the subscale scores provide this information. This recommendation, of using no total score, is in keeping with other widely used coping measures such as the Brief COPE [48].

## Discussion

This research resulted in the development of a 32-item measure of premenstrual coping containing five subscales. Initial psychometric testing of the PMCM provided support for its reliability and validity, although further assessment of these properties, in future research, is recommended. This should include assessing test re-test reliability of the measure. The five factor structure derived from the process of factor analysis represented most of the conceptually developed categories that were observed during the generation of the item pool. This structure was well supported by the open-ended responses which were consistent with previous research on premenstrual coping [34,44,55].

The Avoiding Harm subscale represents the way a woman copes by avoiding situations, people, conversations and thoughts that have the potential to cause distress when she feels more sensitive premenstrually. Past qualitative research [34,55] and the current study’s open responses for coping, identified this as one of the most effective ways women cope premenstrually. This subscale also contains elements of social withdrawal, as avoiding potentially upsetting situations with friends and family may involve a reduction in social interaction. This is in keeping with previous research indicating that women use social withdrawal as an effective means of coping with premenstrual changes [34,39,44], and contradicts claims [36] that social withdrawal and avoidance of situations are maladaptive premenstrual coping processes. Another aspect of the Avoiding Harm subscale, the focus on cognitive structuring, reflected in the item “I challenge my negative thoughts”, is in keeping with research showing the beneficial role of cognitive restructuring in alleviating premenstrual distress [17,19-21].

The Awareness and Acceptance of Premenstrual Change subscale captures the way a woman copes by being aware of both her physical and emotional changes, accepting that they are a normal part of her experience. Past qualitative research has revealed that women benefit from this acceptance as they are less likely to pathologise their premenstrual experience and more likely to engage in other behavioural coping strategies [34]. The identification of this subscale is also consistent with research showing that distancing, which includes acceptance of a stressor [38], is associated with less premenstrual symptom severity [37]. Acceptance of experience is also a quality of mindfulness [56,57] which has been suggested to have relevance in reducing premenstrual distress [34,58] and symptom severity [59].

The Self-Care subscale describes a way of coping which involves a woman focusing on her own physical and emotional needs by engaging in activities which make her feel more comfortable and relaxed. This supports previous research on coping skills interventions which found that increasing self-care during the premenstrual phase had benefits for women experiencing PMDD [20]. The Communicating subscale represents a way of coping based on seeking support and telling others about feelings and needs. The identification of this subscale is consistent with previous findings that women who use social support experience less severe premenstrual symptoms [35,41], and that communication of premenstrual needs and experiences can ameliorate distress [40,60].

The Adjusting Energy subscale incorporates items that demonstrate how a woman copes by adjusting her behaviour to regulate her physical and emotional state, including emotional outbursts, eating sugary foods, and decreasing exercise and involvement with others. The items of this subscale are of particular interest, as the content analysis revealed some women found these items to be the *most* helpful, and others found them the *least* helpful. This indicates that there is a qualitative difference in the use and interpretation of these coping behaviours. Each of these behaviours is associated with discursive representations of idealised femininity [41], suggesting that transgression of the ideal may lead some women to position these strategies as unhelpful. Thus, exercise and healthy eating are positioned as essential in the regulation of the female body, ensuring a slim and toned body shape [61]. Equally, control of negative emotions and attendance to the needs of others are fundamental aspects of the role of *good wife and mother*, with premenstrual transgression of such ideals previously reported to be a source of women’s distress [55,62]. Behaviours such as changes in interest in usual activities, increased food cravings, increased rest, reductions in productivity and being less sociable, are also defined as symptoms in standardised measures of PMS and PMDD such as the PSST, creating a discursive context for some women positioning these strategies as problematic, rather than productive. This demonstrates the need for caution in the use of diagnostic symptom lists which present a purely dysfunctional view of women’s premenstrual behaviours.

A number of coping strategies which were identified in the literature and represented in the original item pool do not appear in the final measure. Active behavioural coping strategies, which include exercising and going out with friends, which have been shown to be helpful for women with PMDD [20], were revealed in the open responses as helpful strategies, but failed to meet cut off points in the factor analysis. Taking pain medication was also identified in the open responses as one of the most commonly listed helpful strategies, yet the original items concerning taking over the counter pain medication and prescribed medication did not form part of the final PMCM. The absence of any medication based coping in the PMCM means that the scale should be considered one which measures psycho-social coping.

The unexpected results from the test for concurrent validity with the one item measure of Dealing with PMS reveal that very few specific coping processes translate simply to a greater perceived ability of coping with PMS as evaluated by this one item measure. Only higher scores on Awareness and Acceptance of Premenstrual Change corresponded to a greater perceived ability to deal with PMS, which indicates that awareness and acceptance plays an important role in premenstrual coping. Communicating was not significantly associated with being able to deal with PMS, and higher scores on Avoiding Harm, Adjusting Energy and Self-Care were all associated with a lower perceived ability to deal with PMS. These results demonstrate that a complex relationship exists between specific premenstrual coping strategies and other aspects of premenstrual experience. The overlap between what is considered coping and what is considered symptoms, discussed above, may account for these mixed results, as these three coping processes stand in contrast to the dominant discourse of the feminine ideal which encourages women to be energetic multi-taskers who nurture others [62] rather than thinking of their own needs. As a result, some women may not interpret Avoiding Harm, Adjusting Energy and Self-Care as coping but instead view these strategies as symptoms, or as failure to cope. Identification with a negative construction of these particular ways of premenstrual coping could thus result in increased feelings of not being able to deal with PMS.

### Limitations, implications and future directions

A possible limitation of this research is the retrospective nature of the data. The participants, unless they were in the 25% of women who were premenstrual when they completed the survey, were recalling past premenstrual changes and coping strategies, which may be considered a threat to validity. However, owing to the recurrent nature of premenstrual experience, the women have had many opportunities to observe their changes and coping behaviours, providing them with a solid basis of experience from which to answer the survey questions. Future research examining premenstrual distress and coping on a prospective basis would be useful, to provide further assessment of the validity of the PMCM scale. Future research could also look at the use of the PMCM with different participant groups to further examine its validity beyond the Australian context, as social context plays a major role in premenstrual experience [63].

The PMCM has significance for use in both clinical and research settings. It may act as a tool for clinicians to use when working with women to both enhance and evaluate premenstrual coping. It also has value for the general population in educating people about premenstrual coping and in helping to promote awareness and understanding of the development and amelioration of premenstrual distress. It offers a step towards a more positive appraisal of some widely experienced premenstrual feelings and behaviours, which have previously been positioned as symptoms. More exploration of why some women and their medical practitioners view certain premenstrual behaviour as symptoms and other women view the same behaviours as coping is warranted. As there is strong evidence that the items of the PMCM offer accessible ways of coping with premenstrual change, it also provides a potential framework to be utilised in future interventions for PMS. For researchers, the PMCM has a number of potential uses including evaluating coping pre and post treatment for moderate-severe premenstrual distress and measuring the relationship between coping and other factors involved in women’s premenstrual experience. Further uncovering of the complex nature of coping in premenstrual experience, found in this study, supports continued research in this area.

## Conclusions

Premenstrual coping manifests as part of the phenomenon of premenstrual change, involving unique ways of coping. This research was able to fulfil the stated aim of developing and providing initial validation of a premenstrual coping measure and has allowed the major psycho-social coping processes relevant to premenstrual experience to be quantified. The development of the PMCM makes a contribution to the field of PMS research, in that future researchers, investigating relationships of variables to premenstrual coping, have the choice of using a specific measure, rather than relying on generic measures of coping which may not tap the unique nature of this construct. The PMCM is available in Additional file 1.

The development of this measure has emphasised the important role of awareness and acceptance of premenstrual changes not only as a coping strategy but as a way for women to gain a sense of agency in being able to cope. It also indicates that helping women to develop more awareness and acceptance of premenstrual change, and an understanding of the negative influences of the dominant discourses regarding PMS, may allow women to access more choices in regard to how they interpret their premenstrual feelings and behaviour.

Rather than developing a diagnostic tool with a focus on symptoms, development of the PMCM has provided a concrete measure of what some women do and others potentially can do to cope with negative premenstrual changes. It has been built on solid understandings that have emerged from qualitative research [34], which acknowledge the role of each woman as expert in her premenstrual experience. A measure of a premenstrual coping that constructs the premenstrual experience beyond ‘symptoms’ or ‘distress’ holds the potential for more positive assessments of premenstrual experience to emerge.

## Competing interests

The authors declare that there are no competing interests.

## Authors’ contributions

JR conceived the scale development, conducted the review of the literature, designed the study, collected the data, performed the statistical analysis and drafted the manuscript. JP participated in the design of the study, advised on development of the item pool, reviewed the statistical analysis, helped interpret the data and draft the manuscript. JU helped with interpretation of the data, drafting the manuscript and revising it critically for important intellectual content. All authors read and approved the final manuscript.

## Pre-publication history

The pre-publication history for this paper can be accessed here:

http://www.biomedcentral.com/1472-6874/14/1/prepub

## Supplementary Material

Additional file 1 **The Premenstrual Coping Measure.** This file contains the PMCM in a ready to use format, with instructions for scoring. Click here for file
