# Supplementary material for: Ways of coping with premenstrual change: development and validation of a premenstrual coping measure
Source: BMC Womens Health. 2014 Jan 3;14:1. doi: 10.1186/1472-6874-14-1 (PMC3880968; doi:10.1186/1472-6874-14-1)
Supplement: Additional file 1 — The Premenstrual Coping Measure. This file contains the PMCM in a ready to use format, with instructions for scoring. [file 1472-6874-14-1-S1.pdf]

The Premenstrual Coping Measure

Women cope with their premenstrual experience in a variety of ways. We are interested in what you do when you are premenstrual. Tick the response choice which indicates how the following statements apply to your premenstrual experience.

**Coping Statements**	**Doesn't apply to me**	**Seldom applies to me**	**Some-times applies to me**	**Applies to me**	**Almost always applies to me**
1. I avoid situations that have the potential to provoke me					
2. I avoid people that have the potential to provoke me					
3. I avoid raising topics that have the potential to create conflict					
4. I remove myself from a situation if it starts to provoke me					
5. I avoid situations where I know I will feel vulnerable					
6. I avoid having conversations that are liable to upset me					
7. I try to avoid dealing with difficult family issues					
8. I challenge my negative thoughts					
9. I accept my changeable moods					
10. I am aware that my premenstrual changes are only temporary					
11. I think it is okay to be feeling differently when I am premenstrual					
12. I am aware of my bodily changes					
13. I think it is okay to be more emotional or sensitive when I am premenstrual					
14. I am aware of my emotional changes					
15. I think that my premenstrual changes are a normal part of a woman's experience					

**Coping Statements**	**Doesn't apply to me**	**Seldom applies to me**	**Some-times applies to me**	**Applies to me**	**Almost always applies to me**
16. I know that other women go through this					
17. I think it is okay that my physical needs may be different					
18. I know what I need to do to support myself					
19. I vent my feelings through emotional outbursts					
20. I decrease my social activities					
21. I focus less on the needs of others					
22. I exercise less					
23. I eat more sugary foods					
24. I spend time doing things that help me relax e.g. have a bath, massage, read a book					
25. I take time to focus on my own needs					
26. I allow myself extra time to rest					
27.I do things to make myself more comfortable					
28. I feel confident to tell people how I feel					
29. I feel confident to tell people what I need					
30. I tell others about how I am feeling					
31. I try not to express how I am feeling					
32. I ask for help from others					

Note to researchers

The PMCM has five subscales representing five premenstrual coping processes. The subscales are:-

Avoiding Harm: Items 1 – 8

Awareness and Acceptance of Premenstrual Change: Items 9 – 18

Adjusting Energy: Items 19 – 23

Self-Care: Items 24-27

Communicating: Items 28 - 32

Scoring is calculated on a 5-point scale with 1 being *Doesn't apply to me* and 5 being *Almost always applies to me.*

No total score is calculated.

Item 31 requires reverse scoring.
